# Integron-Mediated Antimicrobial Resistance and Virulence Factors in *Salmonella* Typhimurium Isolated from Poultry

**DOI:** 10.3390/ani14233483

**Published:** 2024-12-02

**Authors:** Elizabeth Kim, Nora Jean Nealon, Katherine A. Murray, Cydney Jardine, Roberta Magnuson, Sangeeta Rao

**Affiliations:** 1 Department of Clinical Sciences, College of Veterinary Medicine and Biomedical Sciences, Colorado State University, Fort Collins, CO 80523, USA; elizabeth.kim@colostate.edu (E.K.); njnealon@gmail.com (N.J.N.); kamurray401@gmail.com (K.A.M.); cydneyjardine@gmail.com (C.J.); magnusonroberta@gmail.com (R.M.); 2 Department of Diagnostic Medicine & Pathobiology, Shreiber School of Veterinary Medicine, Rowan University, Mullica Hill, NJ 08062, USA; 3 California Department of Food & Agriculture, Sacramento, CA 95814, USA

**Keywords:** poultry, integrons, virulence factors, *Salmonella* Typhimurium, antimicrobial resistance

## Abstract

This study investigates antimicrobial-resistant (AMR) *Salmonella* Typhimurium in poultry, focusing on how class I integrons contribute to AMR and virulence. Using whole genome sequencing, researchers analyzed 26 *S.* Typhimurium isolates from U.S. poultry, finding that three isolates contained integrons (1000 base pairs each). These integron-positive isolates exhibited significantly higher resistance to beta-lactams, phenicols, and tetracyclines compared to integron-free isolates (*p* = 0.004, 0.009, and 0.02, respectively) and harbored genes like *ges*, *imp*, and *oxa*, which are linked to extended-spectrum beta-lactamase resistance. Most AMR gene classes (64%) were chromosome-based, with integron-positive isolates showing a broader array of resistance genes, including *catB* and *tetA*. Integron-bearing isolates had higher occurrences of bacteriocin genes and specific AMR genes like aminoglycoside and beta-lactam resistance genes, while integron-free isolates had more fimbrial and pilus genes. The presence of integrons may trend with increased AMR genes and virulence factors, highlighting the role of integron screening in enhancing AMR surveillance and reducing the need for high-priority antimicrobial treatments in poultry. These findings could support better AMR stewardship practices in poultry production, potentially lowering infection risks in humans and livestock.

## Simple Summary

This study explores antimicrobial-resistant (AMR) *Salmonella* Typhimurium in poultry, emphasizing the impact of integrons on resistance genes and virulence factors. Using whole-genome sequencing, researchers analyzed 26 poultry-derived isolates, finding integrons in three that correlated with greater resistance to beta-lactams, phenicols, and tetracyclines compared to non-integron isolates. Integron-positive isolates also displayed resistance to aminoglycosides and contained high-priority resistance genes, such as *ges*, *imp*, and *oxa*. While AMR genes like *catB* and *tetA* were more common in integron isolates, resistance patterns sometimes appeared in integron-free samples. Integron-bearing isolates had more significant virulence factors like bacteriocin genes, while non-integron isolates showed more fimbrial and pilus genes. These findings suggest that type-I integrons may enhance AMR and virulence in *S.* Typhimurium from poultry, aiding in better AMR surveillance and more effective antimicrobial stewardship in poultry production.

## 1. Introduction

Antimicrobial resistance (AMR) is one of the most recognizable global public health concerns that has an effect on the overall human and animal health [1]. A notable contributor to this escalating concern is the rise in global antibiotic consumption by humans and animals; this is especially the case seen in animal production as the demand for animal protein continues to increase globally [2,3]. Of the widely known AMR pathogens, fluoroquinolone-resistant non-typhoidal *Salmonella* spp. are one of the leading causes of acute gastroenteritis worldwide and are listed as a high-priority threat under WHO global priority AMR pathogens [4]. It was estimated that non-typhoidal *Salmonella enterica* (*S. enterica*) causes approximately 153 million illnesses and 57,000 deaths worldwide [5,6]. In the United States alone, non-typhoidal *S. enterica* causes approximately 1.35 million infections, 26,500 hospitalizations, and 420 deaths annually [7]. *Salmonella enterica* serovar Typhimurium (*S.* Typhimurium) is one of the *S. enterica* serovars most commonly associated with invasive non-typhoidal *Salmonella* disease and has global relevance in relation to livestock and poultry production as it is a zoonotic pathogen known to have a wide range of hosts [8]. One of the hosts that *S.* Typhimurium can be commonly detected is in clinically healthy and diseased poultry, including chickens and turkeys, which are generally raised in large food production systems [9,10]. This poses a risk to food safety as *Salmonella* infection in people and other animals occurs mainly due to the improper handling and consumption of poultry products as well as interactions with fowl that are shedding the pathogen [11].

The prevalence of AMR in *Salmonella* spp. detected in poultry can range from 30% to 40%, with *S.* Typhimurium being a common serotype [12]. With AMR in *Salmonella* being fairly prevalent, this becomes an issue as it not only has an impact on poultry industries and causes large economic losses, but it also results in more severe outcomes of the infection, along with increased hospitalization and deaths [13]. Furthermore, *S.* Typhimurium has acquired new virulence genes that influence host-tropism over the decades, including the acquisition of horizontally transferred genes, which allows for this pathogen to have a broad range of hosts [11]. Virulence factors, such as host adhesion and phagocytosis resistance, can affect the way that *S.* Typhimurium infects a host as well as evade the host immune system, causing a disseminated infection that could escalate and result in severe disease [11]. The combination of the presence of AMR genes in *S.* Typhimurium along with its virulence determinants results in a dual concern in food safety in relation to not only the animals but also people, which include the animal caretakers, meat handlers, and consumers.

The presence of integrons within the *S.* Typhimurium genome is an emerging area of research as it relates to microbial carriage of AMR genes and virulence determinants. An integron is a genetic element that allows mobile gene cassettes to become captured and expressed using site-specific recombination [14]. Among integron types, class I integrons are genetic elements that play a role in integrating gene cassettes from AMR genes into a bacterial genome, contributing to the escalating concern regarding AMR pathogens [15]. A previous study showed how integron-containing *S.* Typhimurium isolates in various host species displayed an increased number and diversity of AMR genes [16]. Although there have been a few studies that showed a relationship between class I integron presence and AMR genes [16,17], there have been limited studies that explored the association of virulence factor genes with class I integrons [18]. Whole Genome Sequencing (WGS) has been used in several studies to identify AMR genes and explore their association with integrons to characterize AMR in different pathogens [19,20]. Using a WGS approach, the primary objective of this study is to examine the association between integron gene presence and carriage of AMR genes and virulence factor genes. Ultimately, the results from this study will be applied to better predict and manage the spread of AMR in *S.* Typhimurium arising from poultry.

## 2. Materials and Methods

### 2.1. Study Design and Sample Collection

This study was designed using the methods described in a previous study by Rao et al., 2020 [16]. To collect samples, multiple United States Veterinary Diagnostics Labs were contacted for *S.* Typhimurium data repositories. Of these veterinary diagnostic laboratories, Colorado State University’s Veterinary Diagnostic Laboratory and the University of Pennsylvania’s Penn Vet Diagnostic Laboratory provided *S.* Typhimurium samples that were isolated from poultry meat birds and collected between 2009 and 2013. A total of 26 samples were donated from these facilities and used in downstream analysis. More details of the sample metadata were unavailable from the source institutions.

### 2.2. S. Typhimurium Isolation and Identification

Each of the 26 poultry isolates was streaked onto trypticase soy agar plates containing 5% sheep blood (Becton, Dickinson and Company, Franklin Lakes, NJ, USA) and incubated overnight at 37 °C. Each isolate was verified to be serogroup B *Salmonella* using traditional slide agglutination (BD Diagnostic Systems^®^, Becton, Dickinson and Company, Franklin Lakes, NJ, USA). The isolated colonies were inoculated into 1 mL trypticase soy broth (Becton, Dickinson and Company, Franklin Lakes, NJ, USA) and incubated overnight at room temperature. After incubation, each isolated colony was mixed with 10% *v*/*v* glycerol prior and stored at −80 °C until downstream testing.

### 2.3. Integron and Antimicrobial Gene Cassette Sequencing

The conserved 5′ and 3′ segments of the class I integron were used as primers to amplify whole integron segments as described in Lucey et al. 2020 [21]. This approach allowed for concurrent sequencing of any antimicrobial resistance and/or virulence genes contained within the integron. The forward primer sequence was 5′-GGC ATC CAA GCA AGC-3′. The reverse integron sequencing was 5′-AAG CAG ACT TGA CCT GAT-3′. Polymerase chain reaction conditions were applied as described in Rao et al., 2008 [16]. Extracted DNA from each isolate was stored at −20 °C until downstream use.

DNA was extracted and purified from excised integron bands using a QIAquick PCR Purification kit (Qiagen^®^, Hilden, Germany). A NanoDrop One spectrophotometer (Thermo Scientific, Lafayette, CO, USA) measured DNA concentrations and validated sample purity and quality and then prepared for sequencing using ABI BigDye ^®^ Terminator v3.1 sequencing chemistry. Forward and reverse integron sequences were processed using an ABI 3130xL Genetic Analyzer (Applied Biosystems™, Thermo Fisher, Foster City, CA, USA).

### 2.4. Sample Processing for DNA Extraction for Whole Genome Sequencing

Twenty-six isolates were thawed in preparation for DNA extraction for WGS. Each isolate was streaked onto blood agar (Thermo Scientific™) and incubated overnight at 37 °C. To confirm sample purity, isolated colonies from each blood agar plate were streaked onto brain heart infusion broth (Thermo Scientific™), incubated overnight at 37 °C, and subjected to DNA extraction using a Qiagen DNeasy extraction kit following manufacturer protocols (Qiagen, Valencia, CA, USA). The concentration, quality, and purity of each sample were verified on a NanoDrop One spectrophotometer (Thermo Fisher Scientific, Wilmington, DE, USA). Sterile extraction media was used as a negative control. Isolated DNA extracts were stored at −20 °C until further use.

The extracted DNA from the 26 poultry isolates was shipped on dry ice to the Animal Disease Research and Diagnostic Laboratory at South Dakota University (Dr. Joy Scaria) to perform WGS as per the previously described methods [22]. DNA concentrations of each sample were normalized to 0.3 ng/µL, given unique barcode identifier sequences via a Nextera XT DNA library prep kit (Illumina Inc. ©, San Diego, CA, USA), and pooled as equi-volume aliquots. Sequencing was performed on an Illumina MiSeq apparatus (Illumina Inc. ©, San Diego, CA, USA) using a 2 × 250 paired-end approach and V2 chemistry.

### 2.5. S. Typhimurium Whole Genome Sequencing Analysis of Chromosomes, Plasmids, and Integrons

A total of 26 *Salmonella* isolates from poultry hosts were included in WGS analyses. Sequencing analysis was performed in Geneious Prime (Version 2020.0.5). A de novo paired-ends approach was used to assemble each genome. BBDuk (Version 38.90) [23] was used to trim index sequences from each isolate. Next, each trimmed isolate was assembled using the Geneious Prime SPAdes plugin (Version 3.13.0), where the assembler method was set to “method = error correct + assemble” and the assembler mode set to “careful mode” using default conditions for these choices [24]. The SPAdes program was also used for plasmid assembly [24].

Each assembled genome and plasmid were screened for the presence of antimicrobial resistance genes and virulence factors via a basic local alignment search tool (BLAST) approach via the publicly available MEGARes 2.0 (antibiotic resistance genes) [25] and Virulence Factor Database (virulence factor genes) BLAST libraries [26,27]. The BLAST parameters used for each isolate and both libraries were as follows: “Match Mismatch = 1-2”, “Gap Cost (Open-Extend) = Linear”, “Max E-value: 10”, “Max Target Sequences = 100” [25]. Integron sequences for each isolate where integrons were present were imported into the Geneious program and BLAST-aligned with the respective isolate chromosomes and/or plasmids to establish their location.

### 2.6. Annotation and Organization of AMR and Virulence Factor Genes

For each *S.* Typhimurium isolate whole genome and/or plasmid, BLAST output was filtered using the following criteria: A positive gene match to a library annotation was defined as a ≥85% pairwise identity match (MEGARes 2.0) or ≥95% pairwise identity (VFDB) of the de novo assembled isolate gene across ≥50% of the reference library gene. Annotations not meeting both of these criteria were removed from downstream analysis. Among the remaining genes, for genes with duplicate annotations, the annotation with the highest pairwise identity match over the longest length and lowest E-value was kept as the reference annotation, and the others were discarded from the analysis. In addition, gene entries were removed if they contained the words “hypothetical”, “tentative”, “possible”, or “predicted” when a duplicate annotation with a confirmed identity was also present. AMR genes were categorized by antimicrobial class, and VFDB genes were categorized by virulence factor function for downstream analysis (Supplementary Materials).

### 2.7. Statistical Analysis

The data on a number of genes were compared between isolates with integrons, and no integrons (integron-free isolates) were compared using a Wilcoxon two-sample test. A Fisher’s exact test was used to evaluate the association of the presence of AMR gene and virulence gene classes with integron presence. All statistical analyses were performed using SAS v9.4 (SAS Institute Inc., Cary, NC, USA), and a *p*-value of <0.05 was used to determine statistical significance.

## 3. Results

### 3.1. Integron Presence and Size

Three of the 26 *S*. Typhimurium isolates from poultry contained integrons, all of 1000 base pairs (bp). Among the 26, 100% carried at least nine AMR genes on chromosomes, and 38.5% (n = 10) carried at least one AMR gene on the plasmid. Among the 26 isolates, 96.2% (n = 25) carried at least 13 virulence factor genes on chromosomes and 80.8% (n = 21) virulence genes on plasmids. The antibiogram of the isolates has been published by Rao et al., 2020 [16].

### 3.2. Integron Presence Impacts Chromosomal and Plasmid Prevalences of AMR Genes in S. Typhimurium

**Figure 1:**
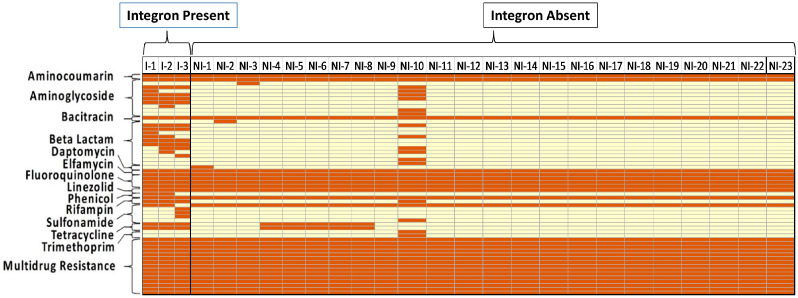
Distribution of antimicrobial resistance genes identified on the chromosome of *S*. Typhimurium isolated from poultry (n = 26) grouped by presence or absence of integrons. Rows are grouped by antimicrobial resistance gene classes. Each column shows the gene profile of an individual *S*. Typhimurium isolates. Each box shows one gene. Red boxes indicate gene presence, and yellow boxes indicate that the gene was not detected in the individual *S*. Typhimurium isolate. Abbreviations: I = Integron Present; NI = Integron Absent.

**Figure 2:**
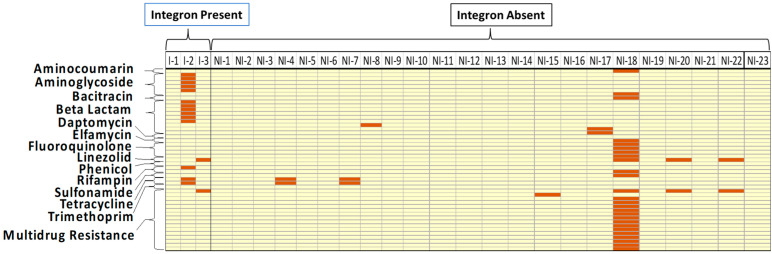
Distribution of antimicrobial resistance genes identified on the plasmid of *S*. Typhimurium isolated from poultry (n = 26) grouped by presence or absence of integrons. Rows are grouped by antimicrobial resistance gene classes. Each column shows the gene profile of an individual *S*. Typhimurium isolates. Each box shows one gene. Red boxes indicate gene presence, and yellow boxes indicate that the gene was not detected in the individual *S*. Typhimurium isolate. Abbreviations: I = Integron Present; NI = Integron Absent.

**Figure 3:**
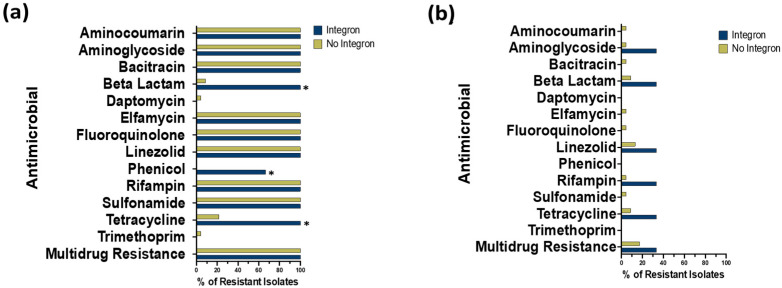
Distribution of antimicrobial resistance gene class prevalences when comparing *S*. Typhimurium isolates with versus without integrons. Blue bars represent isolates with integrons (n = 3), and green bars represent isolates without integrons (n = 23). Panel (**a**) shows gene class distribution prevalences on the chromosome, and (**b**) shows gene class distributions on the plasmid. Prevalences were compared between isolates with and without integrons using Fisher’s exact test, and significance was defined as *p* < 0.05. Significance is denoted with an *.

BLAST analysis of *S*. Typhimurium whole genomes was used to establish the AMR genotypes of all isolate chromosomes and/or plasmids. Figure 1 and Figure 2 show the distribution of AMR genes on the chromosomes and plasmids of *S*. Typhimurium isolates, respectively, with and without integrons, where genes are grouped by AMR gene classes (rows), and gene presence versus absence is shown as a column for each isolate. AMR genes were identified on the chromosome of all 26 *S*. Typhimurium isolates (Figure 1) and identified on plasmids of 10 isolates (Figure 2). When examining the chromosomes of *S*. Typhimurium isolates containing integrons (n = 3), AMR drug classes beta-lactams, phenicols, and tetracyclines were significantly higher (100%, 66.7%, and 100%) (*p* = 0.004, *p* = 0.009, and *p* = 0.02, respectively) compared to isolates with no integrons (8.7%, 0%, and 21.7%). However, when comparing the plasmids of isolates with and without integrons, no significant differences in AMR gene prevalences were identified for any gene class, including fluoroquinolone genes (Figure 3), which is in the WHO priority list [5].

The distribution of individual AMR chromosomal genes was additionally compared between isolates with and without integrons (Figure 1). Within the aminoglycoside genes harbored on the chromosome, the AMR gene *aac(6′)*, which encodes for an aminoglycoside 6′-N-acetyltransferase enzyme, was found within all 26 isolates regardless of integron presence. The aminoglycoside AMR genes *ant(2″),* which encodes for an adenine nucleotide translocase enzyme, and *aac(3)*, which encodes for a 3-N-aminoglycoside acetyltransferase enzyme, were found in the chromosome of all three integron-containing isolates and were absent in all but one of the isolates without integrons. The aminoglycoside AMR gene *ant(3″)*, which encodes for an adenine nucleotide translocase enzyme, was also found within all three integron-containing isolates and was absent in all isolates without integrons. Among beta-lactam genes, the AMR genes *ges* and *imp*, which encode for a Guiana extended-spectrum beta-lactamase enzyme and an imipenemase enzyme, respectively, were found on all three integron-containing isolates and were absent in all isolates without integrons. The beta-lactam AMR gene *oxa*, which encodes for a class D beta-lactamase enzyme, was also found on all three integron-containing isolates but only found in one of the 23 isolates without integrons. Similarly, the beta-lactam AMR gene *carb*, which encodes a class A beta-lactamase enzyme, was found in two of the integron-containing isolates and one of the 23 isolates without integrons. Other notable AMR gene classes found located on the chromosome were phenicol and tetracycline. For phenicol AMR genes, *catB*, which encodes for a type B chloramphenicol acetyltransferase enzyme, was found in two of the integron-containing isolates and absent in all the isolates without integron. As for tetracycline, the AMR genes *tetA* and *tetR*, which encode for tetracycline efflux pumps, were found in all three integron-containing isolates and in five of the isolates without integrons.

When evaluating the AMR genotypes of *S*. Typhimurium plasmids, two of the three *S*. Typhimurium isolates with integrons contained AMR genes on their plasmid (Figure 2). There was a greater difference in carriage of aminoglycoside and rifampin AMR genes between isolates with integron and with no integron (33.3% and 4.35%, respectively, for both genes), although none of the differences were statistically significant.

### 3.3. Integron Presence Impacts Chromosomal and Plasmid Prevalences of Virulence Factor Genes in S. Typhimurium

**Figure 4:**
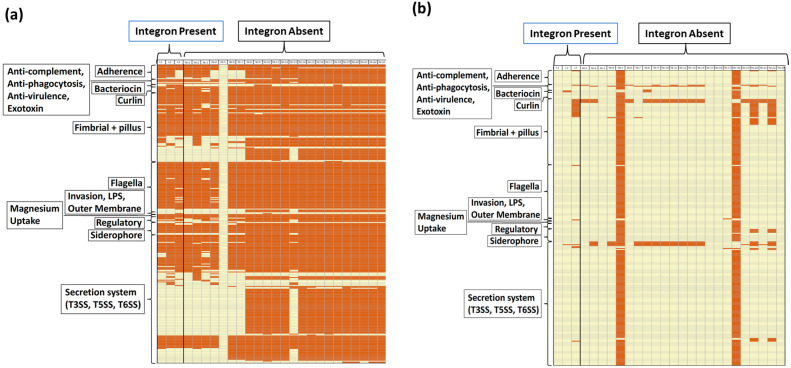
Distribution of virulence factor genes identified on the chromosome (**a**) and plasmid (**b**) of *S*. Typhimurium isolated from poultry (n = 26) grouped by presence or absence of integrons. Rows are grouped by antimicrobial resistance gene classes. Each column shows the gene profile of an individual *S*. Typhimurium isolates. Each box shows one gene. Red boxes indicate gene presence, and white boxes indicate that the gene was not detected in the individual *S*. Typhimurium isolate. Abbreviations: I = Integron Present; NI = Integron Absent.

**Figure 5:**
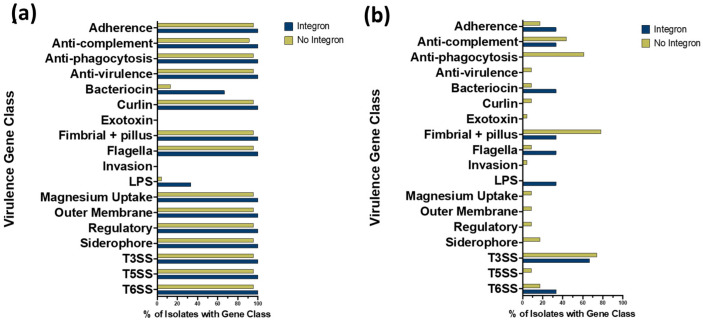
Distribution of virulence factor gene class prevalences when comparing *S*. Typhimurium isolates with versus without integrons. Blue bars represent isolates with integrons (n = 3), and green bars represent isolates without integrons (n = 23). Panel (**a**) shows gene class distribution prevalences on the chromosome, and (**b**) shows gene class distribution prevalences on the plasmid. Abbreviations: LPS = Lipopolysaccharide; T3SS = Type 3 secretion system; T5SS = Type 5 secretion system; T6SS = Type 6 secretion system. Prevalences were compared between isolates with and without integrons using Fisher’s exact test, and significance was defined as *p* < 0.05.

In addition to AMR genes, virulence factor gene type and prevalence were compared on the chromosomes and plasmids of *S*. Typhimurium isolates with and without integrons (Figure 4 and Figure 5). Figure 4 shows the distribution of virulence factor genes on the chromosome (Figure 4a) and plasmid (Figure 4b) of each isolate, where genes are grouped by virulence factor gene classes (rows), and gene presence versus absence is shown as a column for each isolate. Virulence factor genes were more prevalent on the chromosome than on the plasmid for the 26 poultry isolates (Figure 4). When examining virulence factor gene prevalences localized on the chromosome, the isolates with integrons had a higher prevalence of bacteriocin and LPS virulence factor gene classes (66.7% and 33.3%, respectively) than isolates without integrons (13.0% and 4.3%, respectively); however, the difference was not statistically significant (*p* = 0.085 and 0.22, respectively) (Figure 5).

Among virulence factor gene classes identified on the chromosome, most were similarly distributed when comparing isolates with versus without integrons (Figure 4). However, the bacteriocin gene class, which encodes for bacteriostatic and bactericidal proteins and peptides, was significantly more prevalent in integron-containing isolates (Mean ± SE = 1.33 ± 0.67) versus isolates without integrons (Mean ± SE = 0.26 ± 0.14) (*p* = 0.035). Among bacteriocin genes, the *cib* gene (colicin ib) and *pECS88_0104* gene (colicin Ia) were present in two of the three integron-containing isolates and in only three of the 23 isolates without integrons. In contrast with bacteriocin genes, the chromosomal prevalence of the virulence factor gene classes flagella (Mean ± SE = 50.67 ± 0.88 in integron-containing isolates, 51.35 ± 2.38 prevalent in isolates without integrons) and T6SS (Mean ± SE = 13.33 ± 0.33 in integron-containing isolates, Mean ± SE = 23.17 ± 1.63 in isolates without integrons) were significantly lower in integron-containing isolates when compared to isolates without integrons (*p* = 0.043 and *p* = 0.046, respectively) (Figure 4).

When examining the prevalences of virulence factor genes on the plasmids, isolates with integrons had a higher prevalence of virulence factor genes under the gene classes bacteriocin, flagella, and lipopolysaccharide (LPS) (33.3%, 33.3%, and 33.3%, respectively) when compared to isolates without integron (8.7%, 8.7%, and 0%, respectively); however, the difference was not statistically significant (*p* = 0.32, *p* = 0.32, and *p* = 0.12, respectively) (Figure 4b and Figure 5). In contrast, the virulence factor gene class anti-phagocytosis was less prevalent in isolates with integrons when compared to isolates without integrons (0%, 60.9%, *p* = 0.085) (Figure 5).

## 4. Discussion

*S.* Typhimurium is among the most frequently identified *Salmonella* serotypes linked to invasive non-typhoidal *Salmonella* infections, significantly affecting both human and animal health [8]. Additionally, AMR in *S.* Typhimurium remains a pressing public health challenge worldwide. Given that poultry commonly hosts *S.* Typhimurium, it is crucial to address AMR within this reservoir to curb new infection cases in both humans and poultry and to reduce economic losses impacting the poultry industry. Studies show that *S.* Typhimurium and other zoonotic pathogens are prevalent in poultry, where high antimicrobial use promotes AMR development, raising health risks and economic burdens by increasing treatment costs and reducing productivity [28,29]. Studies further highlight the productivity losses in poultry production due to AMR, as well as the risk of resistant strains transferring to humans via the food supply chain. This underscores the critical need for enhanced surveillance and biosecurity measures on poultry farms [30,31]. Additionally, class I integrons, frequently associated with AMR in *S.* Typhimurium across human and livestock populations, warrant further research to understand how integrons influence the carriage of AMR and virulence genes in *S.* Typhimurium to address the rising AMR concerns [16]. The CDC (2022) [32] notes that infections with resistant *Salmonella* strains are often more severe and lead to higher hospitalization rates.

Although numerous studies have characterized AMR in *S.* Typhimurium from poultry [33,34], there is limited knowledge on how integron presence specifically influences the types of AMR genes carried by *S.* Typhimurium in this population, both on the chromosome and plasmids. In our study, we observed that two major AMR gene classes, aminoglycosides and beta-lactams, were more frequently localized on the chromosome in isolates containing integrons compared to those without. Prior studies also demonstrate high resistance levels to these antibiotic classes in *Salmonella* spp. isolated from poultry [35,36,37], suggesting that integrons may be a contributing factor to the persistence of these resistance genes in poultry hosts.

At the gene level, the aminoglycoside resistance gene *aac(6′)* was consistently present on the chromosome in all isolates, regardless of integron presence. Other aminoglycoside resistance genes, such as *ant(2″)*, *aac(3)*, and *ant(3″)*, were found in all three integron-containing isolates but were found in only one of those without integrons. Similarly, for beta-lactam resistance, genes such as *ges*, *imp*, and *oxa* were observed in all integron-positive isolates, while they were infrequent in integron-free isolates. These findings reinforce the potential role of integrons in expanding and stabilizing AMR gene profiles within *S.* Typhimurium in poultry populations.

Two additional notable AMR gene classes, phenicol and tetracycline, were observed on the chromosome in integron-containing isolates compared to those without integrons. Specifically, within the phenicol class, the *catB* gene was present in two of the integron-positive isolates but absent in all integron-free isolates. For tetracycline resistance, *tetA* and *tetR* genes were observed in all three integron-containing isolates, whereas only five integron-free isolates carried these genes. Although the phenicol and tetracycline classes exhibited fewer AMR genes than the aminoglycoside and beta-lactam classes, the consistent association of integrons with specific genes in these categories highlights integrons’ potential role in enhancing resistance profiles within *S.* Typhimurium in poultry.

In addition to the association between AMR genes and integron presence, few studies have examined the relationship between integrons and virulence factor genes in *S.* Typhimurium isolates from poultry. In this study, we observed that the overall virulence factor profile remained similar across most isolates, regardless of integron presence or isolate location. However, bacteriocin genes were an exception. Within the chromosome, bacteriocin genes were more prevalent in integron-containing isolates (2 of 3) compared to those without integrons (3 of 23). Specifically, the bacteriocin-related virulence genes *cib* (colicin Ib) and *pECS88_0104* (colicin Ia) were identified. Colicins, produced by *S.* Typhimurium, provide a competitive advantage by killing susceptible bacteria, thus supporting pathogen survival in a host environment [38].

While few studies have explored colicin prevalence in *Salmonella* from poultry [39,40], some research indicates that colicin expression can influence disease severity by enhancing bacterial competitiveness and fitness in the host, particularly under inflammatory conditions. For example, in *S.* Typhimurium studies, colicin Ib (*ColIb*) expression, intensified by gut inflammation, allowed *S.* Typhimurium to outcompete sensitive *E. coli* strains, potentially worsening disease in animal models. This intensified competition due to colicin may disrupt the microbial balance, contributing to more severe disease outcomes [41].

A study using a commensal *E. coli* model in mice demonstrated that while colicin production may not be crucial for gut colonization under normal conditions, it provides a competitive edge under environmental stressors like inflammation or resource competition. This suggests that colicins play a role in virulence and competitive survival, particularly in microbiomes disrupted by external pressures [42]. These findings imply that colicin expression, although dependent on specific environmental factors, can influence the severity of bacterial infections by shifting microbial populations and enhancing resistance. Investigating its prevalence and clinical implications among non-typhoidal *Salmonella* isolates, including in infections that have integron-containing isolates, consequently merits further investigation.

To date, no studies have specifically examined how the chromosomal virulence factor genes in *S.* Typhimurium are associated with class I integrons, which could help in better characterizing the virulence profiles of *S.* Typhimurium isolates. In our study, we observed no consistent pattern in the localization of AMR or virulence factor genes on plasmids, underscoring the potential importance of chromosomal studies in understanding AMR and virulence determinants in *S.* Typhimurium and their link to integrons. Future research focusing on chromosomal AMR and virulence genes may clarify the role of integrons in these processes, thereby aiding in the development of predictive tools for pathogenicity in *S.* Typhimurium.

There are several limitations to consider in this study. First, the total sample size of 26 isolates, spanning only two different US regions and institutions, may not fully represent the prevalence of S. Typhimurium isolates in other US or global regions. In addition, only three out of the 26 poultry isolates contained integrons, all of which were of a single size (1000 bp), limiting the potential for statistical analyses due to the small sample size. Prior research suggests that the low prevalence of integrons may be influenced by varying environmental factors, such as reduced antibiotic exposure [43]. Additionally, recent improvements in antimicrobial stewardship within the poultry industry may also contribute to the lower occurrence of integron-containing isolates, as many major poultry producers have committed to reducing antibiotic use [44,45].

Further, the 26 poultry isolates were collected from two institutions over a span of four years, which may introduce variability related to geographic and temporal differences in microbial populations. Moreover, it remains challenging to assess how integron size influences the carriage of AMR or virulence genes on chromosomal and plasmid locations, as no studies to date have thoroughly examined this association. Further research on integron size and its impact on AMR and virulence gene carriage is needed to clarify these relationships.

## 5. Conclusions

This study advances our understanding of the complex interplay between AMR genes, virulence factor genes, and integron presence in *S.* Typhimurium isolates from poultry. These insights could aid in addressing AMR concerns by informing the development of early screening tools that better predict *S.* Typhimurium pathogenicity. Such predictive measures would support more effective disease management and potentially guide the creation of targeted antimicrobial therapies to counteract specific virulence mechanisms identified in poultry-associated *S.* Typhimurium.

## Data Availability

Data will be made available on reasonable request.

## Supplementary Materials

The following supporting information can be downloaded at: https://www.mdpi.com/article/10.3390/ani14233483/s1.
